# Extraction of Bioactive Compounds From *Withania somnifera*: The Biological Activities and Potential Application in the Food Industry: A Review

**DOI:** 10.1155/ijfo/9922626

**Published:** 2025-04-25

**Authors:** Siva Karthikeyan Singirala, Praveen Kumar Dubey, Swarup Roy

**Affiliations:** Department of Food Technology and Nutrition, School of Agriculture, Lovely Professional University, Phagwara, Punjab, India

**Keywords:** adaptogens, bioactive compounds, extraction, functional food, phytochemicals, *Withania somnifera*

## Abstract

As awareness of the link between diet and health grows, people are increasingly prioritizing functional foods that offer additional health benefits beyond basic nutrition. Ashwagandha, scientifically known as *Withania somnifera (WS)*, is a perennial plant which belongs to the family Solanaceae, which grows abundantly in subtropical regions of the world. Ashwagandha is a renowned Ayurvedic herb with diverse applications in global dietary supplements and traditional medicines. It has extensive medicinal potential in traditional Indian systems such as (Ayurvedic, Unani, and Siddha) and contemporary medicine, recognized as the “Indian ginseng.” *WS* is a dietary additive composed of various phytochemicals and active compounds such as withanolides, polyphenols, flavonoids, alkaloids, which exhibit therapeutic properties, including anti-inflammatory, anticancer, antistress, antioxidant, antimicrobial, antidiabetic, cardioprotective, hypoglycemic, hepatoprotective, immunomodulatory, and rejuvenating effects. *WS* has been scientifically proven to be highly effective against numerous neurological and psychological disorders. The incorporation of ashwagandha into food enhances the biological activity of the food as well as enhances the functional properties, making it a valuable functional food with potential health benefits. This review provides an updated analysis of *WS*, emphasizing its bioactive compounds, extraction techniques, and functional food applications. Unlike previous studies that primarily focused on its medicinal properties, this review highlights integration into food systems, addressing technological challenges, stability, and commercial viability. Additionally, it compiles advancements in analytical techniques, offering insights into enhancing bioavailability and sensory optimization, thereby bridging traditional herbal use with modern food science.

## 1. Introduction

For centuries, herbs have been used as a natural source of medication for curing a wide range of disorders. In recent years, there has been a renewed scientific focus on incorporating WS into food products, primarily due to its rich profile of bioactive compounds and potential nutritional benefits [1]. WS is widely used in herbal medicine due to its potential health benefits and therapeutic efficacy [2]. Among herbs, ashwagandha, also known as Indian ginseng or winter cherry, is one of the most important herbal plants [3]. This plant belongs to the Solanaceae family, and its scientific name is WS. It has a rich history in traditional ayurvedic treatment for more than 3000 years [4]. It is also known as “Queen of Indian herbs” and is commonly used in Siddha, Unani, and Chinese medicine [5]. WS is a rich source of bioactive compounds, which includes phenolics, flavonoids, withanolides, and alkaloids, which contribute to its therapeutic significance [6, 7]. The total phenolic and flavonoid content varies between different extracts, with WS root extract containing 28.26 mg/g of total phenolic compounds and 17.32 mg/g of flavonoids, whereas WS leaves comprise 5.4 and 5.1 mg/g, respectively. The withanolide concentration also differs across plant parts, with roots containing 0.066%–0.035%, stems containing 0.048%, and leaves containing 0.238%. Additionally, root extracts exhibit withanolide levels ranging from 0.003% to 0.051% in solid form and 0.027%–0.065% in liquid form. Furthermore, research indicates that the total alkaloid content in Indian WS roots ranges from 0.13% to 0.31%, underscoring the phytochemical variability and potential therapeutic applications of the plant [8].

Studies have shown that WS demonstrates a wide range of therapeutic properties, including anticancer, antifungal, anti-inflammatory, antioxidant, and cardioprotective effects [1]. In vivo studies have shown the potential health benefits of WS for enhancing memory, reducing anxiety, and improving lung function in COPD patients, while in vitro studies have shown its ability to inhibit the main protease (MPro) of SARS-CoV-2, indicating a potential role in fighting COVID-19 [6, 9, 10]. The leaves and roots of ashwagandha are primarily utilized for therapeutic purposes and are a rich source of minerals (10.1%) and dietary fiber (28.8%). Additionally, they contain 0.16% of aferine [11]. The nutritional composition of ashwagandha root powder per 100 g contains moisture (7.45%), ash (4.41 g), protein (3.9 g), fat (0.3 g), crude fiber (32.3 g), energy (245 kcal), carbohydrates (49.9 g), iron (3.3 mg), calcium (23 mg), total carotene (75.7 *μ*g), and vitamin C (5.8 mg), making it a valuable component for functional foods [12].

The incorporation of ashwagandha into food products is aimed at utilizing its health benefits, with this functionality and holistic approach motivating consumers to include it in their diet. In previous studies, the powder extracts of WS have been incorporated into different foods such as Shrikhand, which resulted in an overall enhancement of sensory properties as well as increased shelf life by 52 days as compared to traditional Shrikhand [13]. Incorporation of ashwagandha root powder into Indian flatbreads resulted in a low glycemic index (GI) and medium glycemic load (GL), suggesting its potential suitability for diabetic individuals and those seeking better blood sugar management [14]. The impact of WS (WASI) *α*-amylase inhibitor treatment on potato chips resulted in reducing acrylamide formation during heat processing [15]. Addition of ashwagandha into functional RTS beverages enhanced their nutritional quality and significantly improved their antioxidant activity, further boosting their potential health benefits of functional beverages [16], whereas the incorporation of WS root powder into milk resulted in an increase in total solids and carbohydrate percentage and a significant reduction of rennet coagulation time (RCT) [17].

In recent years, there has been a growing interest in functional foods enriched with bioactive compounds to enhance both nutritional and therapeutic benefits. WS is traditionally known for its adaptogenic and medicinal properties, but its application in the food industry remains underexplored. This manuscript provides a novel perspective by compiling the latest advancements in bioactive compound extraction, characterization, and their incorporation into functional food formulations. Unlike previous reviews that focus primarily on its pharmacological effects, this study highlights its integration into dairy, bakery, and beverage products, addressing key challenges such as stability, bioavailability, and sensory acceptability.

**Figure 1:**
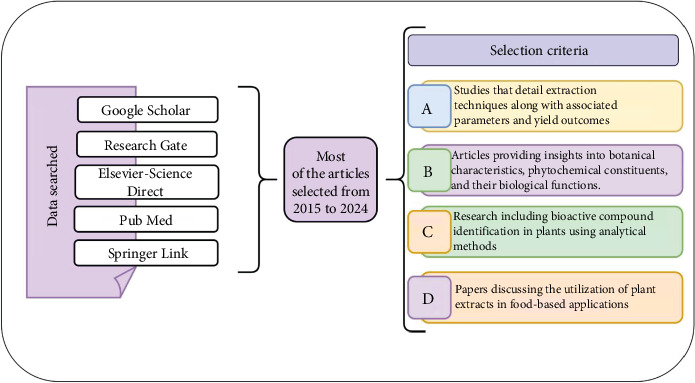
Schematic representation of the systematic literature survey on *Withania somnifera*.

The findings of this review have significant implications for future food innovation, particularly in the development of nutraceuticals, functional dairy products, and plant-based supplements. Understanding the physicochemical properties of WS bioactive compounds will enable better formulation strategies, improved product stability, and consumer acceptance. Additionally, advancements in nanoencapsulation and targeted delivery systems may further enhance its efficacy in functional foods. This review is aimed at providing a comprehensive understanding of the extraction methods, chemical composition, biological activities, and potential applications of WS in developing functional foods. The systematic literature review was conducted using various scientific databases, including Google Scholar, ResearchGate, Elsevier-Science Direct, PubMed, and Springer Link, ensuring a comprehensive collection of relevant studies shown in Figure 1. Most of the selected articles span from 2015 to 2024, providing up-to-date insights into the field. The selection criteria focused on four key aspects: studies detailing extraction techniques and yield parameters, research providing botanical, phytochemical, and biological insights, studies identifying bioactive compounds using analytical techniques, and papers discussing the application of plant extracts in food-based formulations. This structured approach ensured the inclusion of high-quality and relevant literature for the review.

## 2. Chemical Composition of *Withania somnifera*

**Figure 2:**
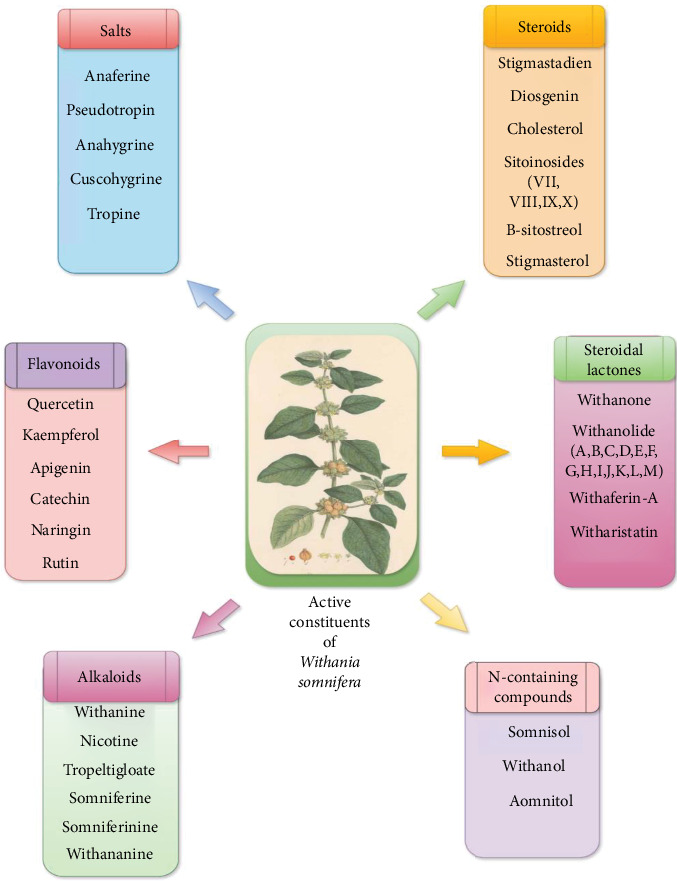
Key active constituents present in *Withania somnifera.*

**Figure 3:**
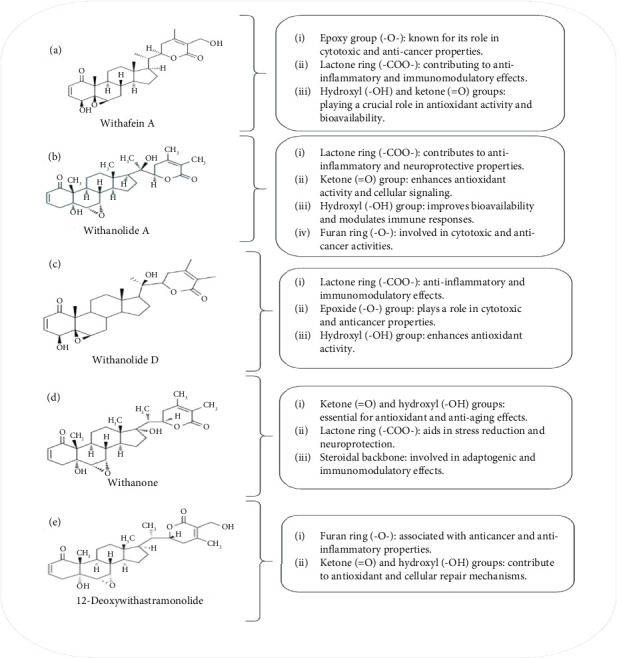
(a–e) Chemical structures of key bioactive compounds from *Withania somnifera* and their functional groups.

WS constitutes a broad range of chemical substances that are found in different parts of plants such as shoots, roots, leaves, fruits (seeds and berries), and bark [18, 19]. These compounds are responsible for various biological actions, including drug formulations, cosmetics, food incorporation, and traditional medicine [20]. The biologically active chemical components of WS are withanolides (0.001%–1.5%) of dry weight (DW), alkaloids (0.13% and 0.31%), and flavonoids (43.51 mg RE/g), which are responsible for therapeutic properties [21]. Among these, withanolides are used in drug formulations due to their therapeutic potential. Numerous studies have been conducted by researchers for the analysis of chemical constituents in the species WS. The isolated compounds are summarized in Figure 2 and Table 1 [1]. To further illustrate the structural diversity and functional significance of WS bioactive compounds, Figure 3 presents the chemical structures of key compounds such as Withaferin A, Withanolide A, Withanolide D, Withanone, and 12-deoxywithastramonolide, highlighting their functional groups (e.g., lactone rings, hydroxyl groups, and epoxides) that mediate anti-inflammatory, antioxidant, and anticancer effects. These structural insights are crucial for understanding the bioactivity and therapeutic potential of WS-derived compounds.

### 2.1. Shoots

In a comprehensive study conducted by [7, 22], various bioactive compounds, specifically withanolides, were identified in the shoots of the plant. These compounds include Withaferin A (3.79 mg/g), Withanolide A (2.88 mg/g DW), Withanolide B (1.48% mg/g DW), Withanolide D (0.30 mg/g DW), and Withanone (0.022 mg/g), along with somniwithanolide, withasomniferanolide, and somniferwithanolide. Each of these compounds significantly contributes to the plant's medicinal properties, as well as a broad range of biological activities and therapeutic applications. The shoots of ashwagandha are well-known for their capacity to regulate hormones, particularly thyroid function and reproductive health [35]. Traditionally, Ashwagandha has been employed to enhance energy levels, combat fatigue, increase vitality, and improve overall well-being due to its adaptogenic properties. These properties help regulate the body's stress response, reduce cortisol levels, and improve mitochondrial function, thereby enhancing endurance and resilience [36]. Furthermore, it has been demonstrated to improve memory, emphasizing its numerous physiological benefits.

### 2.2. Roots

Numerous studies have been conducted on the roots of plants, revealing a wide range of bioactive compounds with extensive medicinal properties. The key compounds identified include Withanolide A (5.4 mg/g DW), Withanolide B (2.59 mg/g DW), Withanolide C, Withanolide D (0.08%–0.11% in DW), Withaferin A (2.36 mg/g DW), withanone (4.32 mg/g DW), Withanolide E, 2,3-dehydrosmonifericin, 2,3-dihydro Withaferin A, 27-hydroxywithanolide A, and withanolide dimer sulphide. Additionally, significant compounds such as 27-deoxy Withaferin A, Coagulin H, Withasomniferol A, Withanoside I (0.0020%), Withanoside II (0.012%), Withanoside III (0.0024%), Withanoside IV (0.048%), Withanoside V (0.017%), Withanoside VI (0.024%), Withanoside VII (0.0011%), 1,2-deoxy-withastranolide (1.12 mg/g), withanine, *β*-sitosterol, and withanone have also been reported in various studies [7, 21, 22]. The roots of WS contribute to a wide range of therapeutic applications. When administered with milk, root powder acts as a potent tonic for children suffering from emaciation; it helps to alleviate age-related frailty, helps manage rheumatism, and relieves constipation [36, 37]. Furthermore, it is used to treat goiter and is applied locally for cold, cough, painful swellings, ulcers, and carbuncles [38]. The roots have the potential to enhance the number of white blood cells, regulate blood sugar levels, and help in weight loss treatment.

### 2.3. Fruits

The fruits of WS constitute numerous bioactive compounds with significant pharmacological activities [39]. Among these, linoleic acid (0.23%), elaidic acid (0.01%), L-asparaginase, fatty acids, sterols, and tocopherols are prominent. Additionally, unique compounds such as 4-deoxywithaperuvin, 14*α*,17*α*-dihydroxywithanolide R, Withanamides A, B, C, D, E, F, G, H, and I have been identified. Other important compounds include hydrocarbons like squalene, tetracosanoic acid (0.880%), and oleic acid (0.14%), as well as 24,25-dihydrowithanolide VI, iso-withanone, and 6*α*,7*α*-epoxy-1*α*,3*β*,5*α*-trihydroxy-witha-24-enolide [1, 25, 29]. These compounds demonstrate the therapeutic properties of fruits present in the WS plant. The fruits of ashwagandha offer a wide range of therapeutic applications, such as acting as anthelmintic, and when combined with astringent and rock salt, they help to remove white spots from the cornea [40]. They also serve as potent diuretics and are also used for treating chest ailments. Additionally, the seeds are used to thicken milk, and berries are used as a substitute for rennet to coagulate milk in cheese making [34]. These findings suggest that the numerous bioactive components in WS fruits have pharmacological importance. However, a more in-depth investigation of their mechanisms and comparative efficacy in therapeutic applications may be beneficial.

### 2.4. Leaves

The leaves of WS, a potent herb, contain a wide range of bioactive compounds that contribute to potential medicinal properties. The key compounds include Withaferin A (1.35 mg/g), 17*α*-hydroxywithaferin A, withanone (1.312%), Withanolide A (350 *μ*g/g), Withanolide B (0.05 mg/g DW), Withanolide C, and Withanolide D (0.08%–0.11% DW). Additionally, compounds like Withanolide E, Withanolide F, WithanosideVI (0.04 ± 0.01 mg/g), Withanoside V (0.11 ± 0.04 mg/g), 12-deoxy withastramonolide (0.07 ± 0.00 mg/L), and deoxywithastramonolide (0.18 mg/g DW) are significant [7, 27]. Further studies have shown the presence of other withanolides and withanosides that enhance the functional properties of the leaves of WS [26]. The leaves of ashwagandha exhibit a wide range of therapeutic applications, such as serving as an anthelmintic (kills intestinal worms) and are recommended for fever, wounds, and painful swelling. The paste made from the leaves is applied locally to eradicate lice on the body, and the leaves are also commonly used at home in the form of herbal tea [34]. WS leaves contain a significant number of bioactive substances such as Withaferin A, along with other withanolides and withanosides, which contribute to their extensive therapeutic properties. These leaves are used in traditional medicine for their antihelmintic, anti-inflammatory, and wound-healing properties, and they are usually taken as herbal tea or used topically.

### 2.5. Bark

The bark of WS contains several important bioactive compounds. Among these compounds are withanolide, withasomnilide, somnifera withanolide, withasomniferanolide, somniwithanolide, and somniferanolide. These compounds contribute to the medicinal properties of the plant's bark, particularly in its traditional use for various therapeutic purposes [27]. Studies have shown that these compounds have potential pharmacological activities and play an important role in enhancing the medicinal value of the bark of WS [41]. The bark decoction of ashwagandha helps to treat asthma, and applying its local application helps with bed sores [42]. The bark of WS contains bioactive substances such as withanolide and somniferanolide, which contribute considerably to its medicinal characteristics and traditional therapeutic applications. These compounds have shown potential pharmacological activity, such as treating asthma and bed sores, highlighting the importance of bark in traditional medicine.

## 3. Alkaloids

WS is a medicinal herb abundant in diverse bioactive compounds, including alkaloids. Alkaloids are one of the important chemical classes found in WS [43, 44]. The isolated compounds are shown in Table 2. These alkaloids play a crucial role in the plant's therapeutic properties, contributing to its various bioactivities, such as anticancer effects [44]. WS contains a range of alkaloids that are synthesized within different parts of the plant rather than being imported, exhibiting the plant's ability to produce these compounds [22]. The alkaloids present in WS contribute to its traditional uses and modern medicine advancement, making it a valuable source of therapeutic agents for various health disorders [18]. The alkaloids present in WS play a significant role in enhancing its therapeutic properties as well as pharmacological significance.

A comprehensive study was conducted on the roots of WS to analyze of the wide range of bioactive alkaloid compounds that contribute to the plant's potential medicinal properties [21]. The key alkaloids identified in the roots of WS include coniine (0.012%), lobeline (0.018%), theobromine, hyoscine (0.015%), yohimbine, ephedrine (0.025%), solanidine (0.018%), somniferinine, withasomine, and somniferine which are well-recognized for their therapeutic effects, which include analgesic, anti-inflammatory, and neuroprotective properties. Furthermore, compounds such as anaferin, isopelletierine, anahygrine, pseudotropine, withanine, scopoletin, nicotine, berberine, caffeine, and theophylline contribute to the plant's medicinal value. These alkaloids show a wide range of biological activities such as muscle relaxation, enhanced cognitive function, mood elevation, and cardiovascular benefits [26, 27]. These compounds demonstrate multiple therapeutic properties and have been utilized in traditional medicine systems [45].

The bioactive alkaloid compounds of WS present in fruits were analyzed by [7]. The fruits of ashwagandha are rich in a unique class of compounds known as withanamides, which enhance the plant's medicinal properties. The key withanamides identified in the fruits of WS include Withanamides A, B, C, D, E, F, G, H, and I. These compounds have strong biological properties, such as anti-inflammatory, antioxidant, and neuroprotective properties. Particularly, withanamides have shown potential to reduce oxidative stress and protect neuronal integrity, which are important for the treatment and prevention of neurodegenerative diseases [27, 45]. The notable alkaloids found in the leaves of WS include vasicine, nicotine, tropine, pseudotropine, tisopelletierine, 3*α*-tigloyloxtropine, anaferin, pseudowithanine, withasomine, 3-tropyltigloate, dl-isopelletierine, withanine, mesoanaferine, somniferin, hentriacontane, visamine (0.014%), withananine, and ashwagandhine [26]. These compounds exhibit various biological activities such as antispasmodic, antiasthmatic, and anti-inflammatory properties. The wide range of alkaloids emphasizes its potential for therapeutic uses in recent pharmacology and enhances its applications in conventional medicine. This further shows the significant medicinal value of the leaves of WS [21, 22]. The presence of diverse bioactive alkaloids in WS significantly enhances the plant's medicinal properties. However, further investigation is needed to optimize the extraction of alkaloid compounds from WS.

## 4. Flavonoids

Flavonoids are the group of bioactive compounds which are found abundantly in WS [55]. The flavonoid content present in leaves, stem, and roots is noted as 43.51 ± 0.346 mg/g, 42.82 ± 1.189 mg/g, and 39.13 ± 0.607 mg/g, respectively [56]. Research has shown that flavonoids have a wide range of health benefits, including lipid metabolism, potentially treating depression and anxiety, improving insulin sensitivity, and enhancing glucose metabolism [57]. The various flavonoids isolated from various parts of the plant are shown in Table 2. Overall, flavonoids present in WS offer a wide array of health benefits and therapeutic potential.

### 4.1. Whole Plant

The study was conducted on the whole plant of WS to identify and analyze the flavonoid compounds. The key flavonoids identified in the plant include naringenin, naringin, and rutin. These compounds are well recognized for their potential biological activities such as antioxidant and cardioprotective properties contributing significantly to the plant's overall therapeutic benefits [7, 50]. The presence of flavonoids enhances the medicinal value of the WS, making it a versatile agent in traditional and modern medicinal applications. Furthermore, these flavonoids play an important role in pharmacological applications [58]. The roots of WS are rich in a wide range of flavonoids, enhancing the plant's medicinal properties. The key flavonoids found in the plant of WS include hyperoside, aesculentin, kaempferol (0.195%), dihydroxykaempferol, catechin, rhamnetin, quercetin, quercetin-3-rutinoside, rutin (0.134%), quercetin 3-O-robinobioside-7-O-glucoside (0.157%), and myricetin; these compounds are known for their potential biological activities by enhancing the therapeutic potential of the roots [26, 47, 48]. The presence of a diverse range of flavonoids in WS significantly enhances the plant's medicinal properties by contributing antioxidant, cardioprotective, and other pharmacological benefits in both traditional and modern medicine.

The aerial parts of the WS contain important flavonoid glycosides that contribute to their medicinal properties. The key compounds identified in the chemical analysis include kaempferol 3-robinobioside-7-glucoside and quercetin-3-rutinoside-7-glucoside. These flavonoids are known for their potential biological activities such as anti-inflammatory and anticancer properties, which enhance the overall therapeutic potential of the plant's aerial components. These components play a significant role in pharmacological applications as well [51]. The berries of WS contain bioactive flavonoid compounds which include apigenin, apigenin-7-O-*β*-D-glucopyranoside, catechin, rutin, and kaempferol, which are recognized for their antioxidative and anti-inflammatory properties. These compounds show potential therapeutic properties [52]. The fruits of WS contain key flavonoid compounds like catechin, kaempferol, and rutin, which have been identified for the chemical analysis of flavonoids [49]. These findings highlight their role in enhancing the medicinal properties of fruits of WS. Similarly, ashwagandha leaves contain important flavonoids such as catechin (0.102%), rutin, 6,8-dihydroxykaempferol-3-rutionside, and quercetin-3-rutinoside-7-glucoside. These compounds contribute to their biological activities [26, 54]. The results shown emphasize the essential functions of flavonoids in the various parts of WS enhancing its overall therapeutic performance.

## 5. Extraction Process

**Figure 4:**
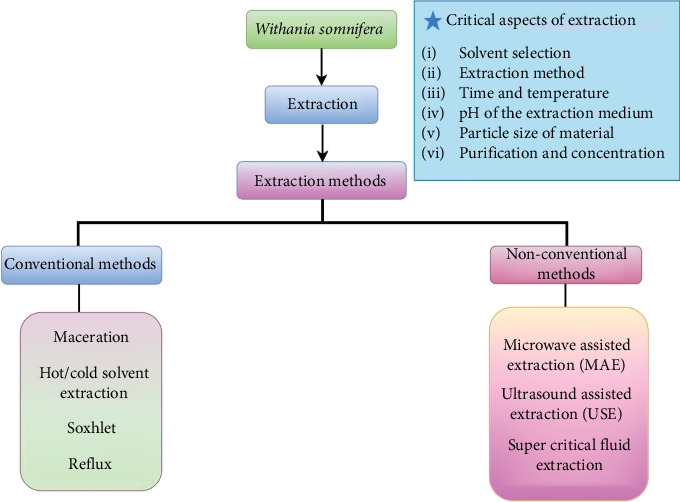
Different methods of extraction process used for ashwagandha.

Extraction is a crucial step in identifying and analyzing bioactive compounds present in natural sources and is a critical aspect of modern research. Extraction techniques are employed to isolate these compounds, enabling their potential benefits to be discovered. It is important to recognize the significance of these compounds, as they have the potential to revolutionize various industries, including medicine and food [59]. The selection of a suitable extraction technique plays an important role, as multiple factors will affect the yield of bioactive compounds based on the type of extraction process [60]. The plant WS possesses multiple therapeutic properties exhibited by the variety of bioactive compounds, like withanolides, alkaloids, and flavonoids that give it its healing abilities [61]. Different extraction methods are shown in Figure 4, which are used to extract these compounds from the plant material, and they are crucial for obtaining the bioactive compounds from WS. Selecting the appropriate extraction method for isolating bioactive compounds from WS relies on several factors, such as the type of compounds, desired extract purity, and intended application. Both traditional and alternative extraction techniques can be utilized for this purpose [7].

Traditional methods for extracting chemical compounds from medicinal plants involve maceration (MC), infusion, reflux, and Soxhlet's extraction (SE), all of which focus on the process of solvent penetration into plant cells, dissolving phytochemicals within the plant structure and extracting phytoconstituent from the cells. Nonconventional extraction methods, such as microwave-assisted extraction (MAE), ultrasound-assisted extraction (UAE), and supercritical fluid extraction (SFE), are also utilized for this purpose [7]. Alternatively, ultrasound-assisted solvent extraction (UASE) is a method that does not involve heat but instead uses high-frequency sound waves to break down plant cell walls, facilitating the extraction of bioactive compounds [62]. This technique has allowed researchers to isolate bioactive compounds from WS, which have potential therapeutic applications.

### 5.1. Conventional Methods of Extraction

#### 5.1.1. MC Extraction

Soaking the herb in a solvent for an extended period is called MC extraction [63]. The active ingredients in the herb are progressively dissolved by the solvent during this period. MC extraction is an effective technique that preserves the sensitive phytochemicals and antioxidants present in plants [64]. As a result, it is being employed for WS extraction. This method is particularly useful for processing sensitive herbs and extracting nonheat-stable components [65]. However, research has revealed that WS cultivated in the winter contains more withanolide content than those shrubs grown in the summer. Modern approaches, such as SFE and pressured liquid extraction, have gained favor due to their great efficiency, self-sufficiency, and cost effectiveness. These extraction techniques have not only yielded better results but also represent advanced methods of performing these extraction processes [63]. This extraction method requires low investment, is convenient to use, and operates through an automated system. However, they are not ideal for heat-sensitive compounds as it involves a prolonged extraction time and high solvent consumption [66].

#### 5.1.2. Solvent Extraction

One method that works well but takes a significant amount of time to extract different bioactive compounds from WS is the SE. It is also known as the continuous hot percolation process. To extract compounds from the leaves, the SE is combined with n-hexane defatting and diethyl ether liquid partitioning, followed by condensation and recycling back into the boiling chamber [67]. Since solvent extraction can readily be scaled up for larger batches and extract the necessary components quickly, it is a common approach for extracting WS. In addition to providing batch-wise or continuous extraction setups as needed, it recycles the extraction solvent. The solvent can extract the active ingredients from the herb by repeating this procedure multiple times. This approach is quite common in large processing platforms due to its scalability and economic sustainability, even if it requires a longer cycle to complete. It guarantees the full extraction of active compounds from the plant matrix, which is another important advantage [59, 68]. This extraction method is convenient to use and operates through an automated system. However, it is not suitable for heat-sensitive compounds, as it requires a longer extraction time and higher solvent consumption [66].

### 5.2. Nonconventional Methods of Extraction

#### 5.2.1. SFE

SFE is the most effective extraction technique that yields an extract with an elevated level of purity. Supercritical fluids, such as carbon dioxide, are used as a solvent in SFE [69]. In one study, it was observed that pulverized and dried WS seeds were extracted with food-grade liquid carbon dioxide and used backflow pressures of 450/80 and 555/40 bar/°C while maintaining a CO2 flow rate of 60 g/min for a long period of 22 h and 20 min, which resulted in a high yield of fatty acids (13%) [70]. Subcritical water extraction (SWE) is regarded as an effective, environmentally friendly alternative approach for extracting bioactive chemicals that yield greater extract yields (EYs) in plant solid sample forms [71]. The primary mechanism in SWE is hydrogen bond weakening and polarity shifts. Because supercritical fluids combine the special qualities of liquids and gases, they are the perfect solvents for removing the bioactive ingredients from WS [72]. SWE was performed with different temperature ranges between 100°C and 200°C, respectively, and the best results were found at *T* = 200°C, *P* = 100 bar. This was compared with conventional methods, namely, MC, SE, and MAE. The highest extraction efficacy was achieved by raising the temperature to 200°C in 30 min using SWE as an alternative method. On the contrary, extraction at 160°C for 20 min resulted in a high concentration of bioactive compounds with significant biological activities. On the other hand, MC, solvent extraction, and MAE displayed much less performing yields of 20.8%, 25.7%, and 30.2%, respectively [72]. These results demonstrate that SWE not only allows for shorter extraction times compared to conventional methods but also maximizes the extraction of beneficial compounds. This extraction method eliminates the need for filtration after extraction, is nontoxic, and operates through an automated system. However, there is a possibility of system blockage and the potential loss of volatile analytes [66].

#### 5.2.2. MAE

The quick and effective technique known as MAE heats the solvent and facilitates the extraction of bioactive compounds from WS [73]. Although MAE is a relatively new method, its yield and extract purity have shown encouraging results [74, 75]. In one investigation, methanol was used to macerate shade-dried and crushed WS leaves, stems, and roots for 14 days. The methanol EYs were higher than those of the extract from the stem and root, which showed entirely different phytochemical profiles with flavonoid content amounting to 43.51 ± 0.346 mg/g in leaves, while that in stems and roots is 42.82 ± 1.189 mg/g and 39.13 ± 0.607 mg/g, respectively [56]. Another significant study reported the isolation of an antiadipogenic withanolide from WS roots of WS using reflux extraction with 80% aqueous methanol [76]. At room temperature, homogenization with methanol–water (8:2), the extracts obtained from the dried root and fruits of WS are 19% and 22%, respectively. In another study, the root raw material was using both conventional methods, such as reflux extraction and SE, and nonconventional methods, such as MAE and UAE. The extracts were assessed for their total phenolic content (TPC), and the overall phenolic content was higher in MAE and UAE compared to those prepared using conventional methods. The findings suggest that the superior penetration and rapid extraction rates in UAE and MAE contribute to the effective and faster extraction of bioactive compounds compared to the conventional techniques [75]. Additionally, MAE offers a more hygienic method of removing bioactive compounds from plant matrices. The technique's main prerequisite is a proficient operational setup. This extraction method offers a short extraction time, high yield, better selectivity, and cost-effectiveness. However, it may lead to potential compound degradation, requires high equipment costs, and involves the use of organic solvents [77].

#### 5.2.3. UASE

UASE is a nonthermal extraction technique that breaks down the cell walls of plant material with high-frequency sound waves to help release bioactive compounds [78]. The plant material is exposed to ultrasonic vibrations while submerged in a solvent, such as water or ethanol [79]. High yields of bioactive chemicals can be extracted quickly and effectively using ultrasonic extraction, which produces these compounds [80, 81]. In one study, WS powdered root raw material was extracted utilizing techniques such as reflux extraction, UASE, and microwave-assisted solvent extraction (MASE). Various extraction solvent proportions were used, including water and ethanol–water mixtures (9:1). UASE and MASE were extracted for 5, 10, and 20 min, respectively, whereas reflux extraction required 5 h. When compared to MASE (13.74%) and UASE (11.85%), the traditional extraction approach (9.51%) has a much lower yield. UASE ethanol extracts (8.66 *μ*g/mg) and MASE ethanol extracts (5.73 *μ*g/mg) contained higher total withanolide content than standard ethanolic extracts (4.79 *μ*g/mg) [59]. This method requires minimal fossil energy and has a low investment cost. However, ultrasound may negatively affect active constituents, and the process requires a large volume of solvent and extensive filtration [82].

Using WS leaves and roots as raw material, two conventional extraction methods (MC and SE) were compared to two modern extraction methods (MAE and SWE). Compared to older processes (MC (20.8%) and SE (25.7%)), new approaches exhibit greater extraction yields (MAE (30.2%) and SWE (65.6%)) and higher bioactive compound content [72]. While methods like MASE and UASE have shown encouraging results, compared to the traditional extraction mode, they are not as economically or practically viable for large-scale operations. More advancements are required to optimize these techniques and make them feasible for large-scale applications. Supercritical extraction is another promising platform that yields clean extracts by minimizing the amount of solvent needed.

## 6. Biological Activities of Ashwagandha

**Figure 5:**
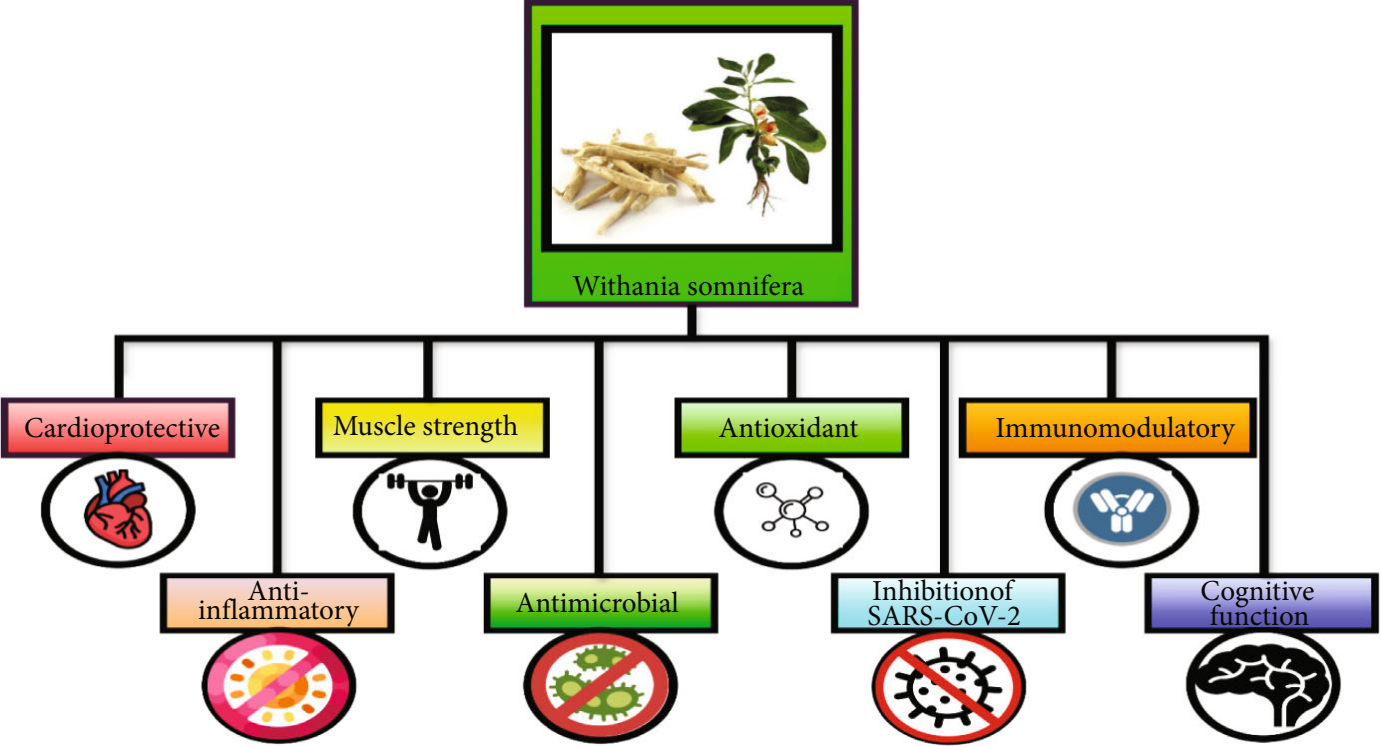
Various biological activities of *Withania somnifera.*

Ashwagandha exhibits a broad range of biological activities; some of these activities include anti-inflammatory, immunomodulatory, antimicrobial, cardioprotective, antioxidant, muscle growth enhancement, cognitive function improvement, and inhibition of SARS-CoV-2 effects [83, 84]. The different biological activities are shown in Figure 5. The bioactive compounds of the plant have been studied extensively for their health properties, demonstrating their potential in the healthcare industry, emphasizing their significance in both traditional and modern medicine [1].

### 6.1. Anti-Inflammatory Properties

WS acts as a natural anti-inflammatory steroid due to its flavonoid content which is responsible for the strong anti-inflammatory effect [85]. Flavonoids have free radicals scavenging activity [86], which regulates the activities of inflammation-related cells, inhibits T cell proliferation, regulates the activity of the enzyme's cyclooxygenase, phospholipase A2, and lipoxygenase, and lowers the formation of arachidonic acid, prostaglandins, and leukotrienes, which are the mediators of inflammation [87]. In another study, the anti-inflammatory properties of ashwagandha were investigated by various experimental studies [12]. Due to the presence of various bioactive compounds in WS, it has shown significant anti-inflammatory activity in both in vitro studies and various models [88, 89]. This shows the WS plant's ability to inhibit key inflammatory substances, including histamine, prostaglandins, and serotonin (5-HT) [90].

The anti-inflammatory properties of WS involve suppressing the MAPK and NF-*κ*B pathways, by modifying the cytokine expression in HaCaT cells and changing mRNA expression levels. Through this mechanism, the anti-inflammatory cytokine TNF-*β* increases specifically, while the proinflammatory cytokine TNF-*α* decreases, which improves its properties in wound healing [91]. These findings support the traditional Ayurvedic use of WS as an anti-inflammatory (Sothaghna) and wound-healing (Vranaropana) agent [12]. The effectiveness of WS in treating different rheumatologic conditions may be due to its anti-inflammatory properties. Studies conducted on the administration of powdered root of WS orally 1 h before being exposed to an inflammatory substance for 3 days showed anti-inflammatory effects like those of hydrocortisone sodium succinate [92]. Additionally, a considerable decrease in nitric oxide production in the presence of lipopolysaccharide (LPS) was observed with 47% methanolic extract of *A. indica*, and its IC50 value was noted to be approximately 33.3 *μ*M as measured by RAW 264.7 cells, which also inhibited TNF-*α* production at equivalent concentration; its IC50 being around 40.9 *μ*M, which confirmed that the anti-inflammatory effect was mediated by a reduction in inducible NOS expression as shown by Western blot analyses, suggesting a transcriptional inhibition [93]. In a similar study, the aqueous extract of WS root was administered to individuals at a dose of 300 mg/kg body weight, resulting in significant enhancement in interleukin-10 (IL-10) secretion as well as nuclear factor-kappa B (NF-*κ*B) activity [94]. The flavonoids present in WS significantly decrease inflammation by scavenging free radicals and blocking critical inflammatory pathways, confirming their ancient Ayurvedic usage for inflammation and wound healing.

### 6.2. Antioxidant Activity

The antioxidant properties of WS are mediated through a wide range of mechanisms, demonstrating its conventional medical uses and aiding in biological mechanisms that fight oxidative stress [18]. One key mechanism involves the control of endogenous enzymatic systems, which fortify the body's defenses against reactive oxygen species (ROS). Furthermore, the bioactive compounds of WS, particularly withanolides, actively fight against free radicals to protect cells from oxidative damage [95]. WS is efficient in reducing oxidative damage, as evidenced by research showing that supplementing with it enhances inflammatory responses and lowers oxidative stress markers. Its antioxidative ability is further enhanced by its potential to modulate hormone levels, which makes it extremely useful in the treatment of chronic illness [23, 96]. This comprehensive understanding of its antioxidative mechanisms underscores the significance of WS in modern health sciences. Furthermore, it was found that WS can scavenge the oxidative free radicals found in brain and nervous system tissues that are enriched in iron and lipid contents, which play a significant role in the production of ROS. These are regulated by the expression of multiple antioxidant enzymes, such as superoxide dismutase (SOD), glutathione (GSH) peroxidase (GPx), and catalase (CAT) in the brain [97]. The leaves of WS are a rich source of antioxidants due to the presence of naturally occurring phenolic and flavonoid compounds [98]. Maximum radical scavenging activity was found in MC extraction compared to the reflux extraction method. Methanol extraction yielded the highest phenolic content (198.24 ± 1.16 mg GAE/g) and flavonoid content (31.52 ± 0.91 mg QE/g), along with the highest DPPH radical scavenging activity (81.98 ± 0.49%). Furthermore, acetone extraction provided the best hydrogen peroxide scavenging activity (76.18 ± 1.06%). These findings suggest that methanol is the most effective solvent for the extraction of antioxidants from the leaves of WS, highlighting its potential antioxidant activity which can be used in therapies [68].

Another study demonstrated that WS enhanced the levels of three natural antioxidants in the brain: GPx, SOD, and CAT [39]. These results support the use of ashwagandha in Ayurvedic medicine as a rejuvenating tonic. The antioxidants in ashwagandha roots can reduce stress, combat aging, improve cognitive function, and effectively fight inflammation in model studies and clinical situations [99]. The potent antioxidant properties of WS are due to the presence of a wide range of bioactive phytochemicals such as steroidal lactones, withanosides, withanolides, flavonoids, and phenolics found throughout its bark, stem, leaves, and roots [100]. However, it was reported that the difference in temperature selectivity during SWE significantly impacted the antioxidant activity and concentration of bioactive compounds like flavonoids and phenolics [101]. This study also supports these findings. Previous research indicated substantial variability and significant differences in ashwagandha extracts, as well as in their biological activity, depending on the type of extraction method used [59]. The findings suggest that WS possesses strong antioxidant potential, particularly in the brain, attributed to its rich phenolic and flavonoid content, and that the extraction technique and temperature play a critical role (specially methanol extract appeared most effective solvent) endorsing its traditional usage to combat oxidative stress and aid healthy cognitive function as per Ayurvedic medicine.

### 6.3. Immunomodulatory Activity

Immunomodulators are substances that modify immune responses by modulating, stimulating, or suppressing the components of the adaptive and innate immune system [102]. WS acts as an effective immunostimulant [85], enhancing both the humoral and cell-mediated immune response [103]. The immunomodulatory properties of ashwagandha root powder inhibit several inflammatory mediators that have been exhibited, including excessive complement activation, the humoral antibody response, and T-lymphocyte proliferation [104]. The role of ashwagandha in mitigating arthritis, an autoimmune disease characterized by elevated inflammatory markers like interleukin-6 (IL-6), IL-10, and TNF-*α*, has also been assessed [105]. Oral administration of 300 mg/kg of ashwagandha is sufficient to decrease the proinflammatory cytokines associated with arthritis such as TNF-*α*, IL-1*β*, and IL-6 in vivo [94]. This immunomodulatory effect of ashwagandha is attributed to chemical constituents such as flavonoids, lactones, and steroids (withanolides) [106].

Another study investigates the immunomodulatory properties of WS on human neutrophils using a series of in vitro analyses. The compounds isolated from the roots of WS were evaluated for their potential to modulate immune functions using the nitro blue tetrazolium (NBT) test, phagocytosis of killed *Candida albicans*, and assessment of neutrophil locomotion and chemotaxis. The compounds were evaluated at varying concentrations: 10, 20, 40, 100, and 1000 *μ*g/mL. The findings illustrated that the isolated compound significantly influenced immune parameters in human neutrophils, thereby indicating a prominent immunomodulatory effect [107]. Furthermore, the extracts from the roots and leaves of WS, standardized for withanolide glycosides (withanoglycosides), can enhance innate and adaptive immune responses in humans. Specifically, these extracts were found to increase the levels of immunoglobulins, T-cells (CD3+ and CD4+), and interferon-gamma (IFN-*γ*), indicating a broad improvement in immune function. These findings suggest that WS extracts can play a significant role in strengthening the immune system's ability to identify and fight against bacteria, viruses, and allergens. This aligns with previous studies conducted on living organisms, which have reported increased expression of T-helper 1 (Th1) cytokines and higher counts of CD4+ and CD8+ T-cells, as well as enhanced natural killer (NK) cell activity. These findings are supported by a study that exhibited a significant increase in CD3+, CD4+, CD8+, CD19+, and NK cell counts after 30 days of treatment with WS extract [108]. The findings suggest that WS can modulate immune responses as well as inhibit several inflammatory mediators.

### 6.4. Antimicrobial Activity

WS exhibits a range of antimicrobial properties that include both direct and indirect actions on microbiological infections. The plant's rich composition of bioactive constituents, specifically steroidal lactones, and alkaloids, which have been demonstrated to have strong antibacterial properties, is an important feature. These substances hinder bacterial growth and multiplication by breaking down microbial cell walls or by blocking vital metabolic functions [18]. Furthermore, by preventing oxidative stress, which is essential for microbial viability, WS's antioxidative qualities increase its antibacterial activity. In addition to enabling direct antimicrobial actions, this dual action strengthens the immune system, enhancing the body's resistance to infections [109]. These diverse mechanisms establish WS as a potent therapeutic agent for addressing microbial resistance and promoting overall health. Despite limited scientific understanding of its mechanism, practitioners of Ayurvedic medicine have traditionally used ashwagandha to combat infections. Recent studies have shown that the methanolic extract of ashwagandha leaves, when applied at a concentration of 2 mg/mL (100 *μ*L per well in an agar well diffusion assay), exhibits significant antimicrobial properties. This extract effectively inhibited the growth of Gram-positive bacteria, including *Enterococcus species* and Methicillin-resistant *Staphylococcus aureus* (MRSA) isolated from the pus samples, exhibiting its potential as a strong antimicrobial agent [110]. Furthermore, it has shown promising antimicrobial activity against various Gram-negative bacteria. These include *Klebsiella pneumoniae*, *Escherichia coli*, *Salmonella typhi*, *Citrobacter freundii*, *Proteus mirabilis*, and *Pseudomonas aeruginosa*. These results suggest a wide range of antimicrobial potential of the ashwagandha extract [49, 111, 112]. In a separate in vitro study, WS stem extracts demonstrated significant antimicrobial activity at a concentration of 100 mg/mL. The extracts exhibited significant efficacy in inhibiting a wide range of pathogens, including *Trichoderma viride*, *Bacillus cereus*, *Aspergillus niger*, and *Serratia marcescens* [113].

A further study found that administering WS root extract at a concentration of 20 ml/L of water significantly decreases the severity and mortality associated with *Escherichia coli* infection [114]. The treatment also improves both humoral and cellular immune responses. Additionally, WS effectively inhibits the infectious bursal disease virus in vitro, exhibiting its potential as an antiviral agent [115]. Another study suggested the potential antibacterial activity of WS against multidrug-resistant strains [116]. Most of the research on the antimicrobial properties of WS extracts and their bioactive compounds has utilized the disc diffusion method. While this method provides an initial understanding of antimicrobial effectiveness, it has inherent limitations. Therefore, it is necessary to complement disc diffusion studies with minimum inhibitory concentration (MIC) assays to obtain a more comprehensive understanding of antimicrobial strength and the precise concentration required to inhibit microbial growth [117]. The leaves of WS have potential antimicrobial properties that can inhibit the growth of Gram-positive bacteria, including *Enterococcus species* and MRSA.

### 6.5. Cardioprotective Activity

WS exhibits its cardioprotective effects through various physiological and biochemical mechanisms, contributing to its traditional and therapeutic role in cardiovascular health. The antioxidant defense mechanism of WS enhances the activity of endogenous antioxidant enzymes, including SOD, CAT, and GPx, which protect the myocardium from oxidative stress-induced damage [118]. In isoprenaline-induced myocardial necrosis models, WS has been shown to significantly reduce GSH depletion and lipid peroxidation, thereby preserving cardiac cell integrity [83]. Furthermore, the hypoglycemic and hypocholesterolemic properties of WS contribute to improved cardiovascular health by lowering blood glucose, total cholesterol, low-density lipoprotein (LDL), and triglycerides while enhancing high-density lipoprotein (HDL) [96]. These effects are attributed to the modulation of glucose metabolism and lipid profile regulation, which reduce the risk of atherosclerosis and metabolic syndrome [21]. Additionally, anti-inflammatory and endothelial protection effects of WS suppress proinflammatory cytokines such as tumor necrosis factor-alpha (TNF-*α*) and IL-6, which are implicated in endothelial dysfunction and atherosclerosis. By mitigating vascular inflammation, WS helps maintain endothelial integrity and prevents the progression of cardiovascular diseases [25]. Cardiovascular performance enhancement in clinical trials has demonstrated that WS supplementation improves cardiovascular endurance, oxygen utilization (VO₂ max), and muscular coordination. This is likely mediated through its adaptogenic and ergogenic properties, enhancing neuromuscular function and circulatory efficiency [119]. WS exhibits diuretic effects by increasing urine sodium excretion and total urine volume, which may contribute to better fluid balance and blood pressure regulation. This mechanism supports its potential role in hypertension management and cardiac workload reduction [21]. Through these interconnected mechanisms, WS serves as a potent cardioprotective agent, mitigating myocardial stress, regulating metabolic parameters, and improving overall cardiovascular function.

### 6.6. Enhancement of Cognitive Function

Research suggests that WS can enhance memory, cognition, and overall brain health [120, 121]. It has been found to enhance cognitive tasks, executive function, attention, and reaction time in people with mild cognitive impairment and other cognitive disorders [121]. Furthermore, WS promotes neurogenesis, making it an important memory enhancer [122]. Several preclinical and clinical investigations have exhibited that WS improves cognitive function in individuals with neurodegenerative disorders, anxiety-induced cognitive dysfunction, and similar conditions [39, 121, 123]. A daily dosage of 100 mg/kg of WS root extract exhibited protective effects against memory loss induced by propoxur, as well as reduced acetylcholinesterase activity in the brain and blood [6]. In another study, oral treatment with 100 mg/kg/day of root extract of WS for 21 days helped prevent cognitive dysfunction caused by bisphenol. The extract's potential to control the activity of antioxidant enzymes such as CAT and SOD as well as to scavenging ROS has been associated with its protective function. Additionally, the extract restored NMDA receptor activity, which had been reduced by bisphenol. This restoration is a key factor in maintaining cognitive function [124]. Reduced activity of acetylcholinesterase has been linked to memory loss and cognitive dysfunction.

### 6.7. Inhibitory Effects of SARS-CoV-2

Several investigations have suggested WS as a potential therapeutic agent for COVID-19 due to its diverse pharmacological properties, including immunomodulation, inflammation regulation, organ protection, antistress effects, suppression of proinflammatory cytokines, and antihypertensive and antidiabetic activities [83]. These properties suggest that WS may serve as a supportive treatment for COVID-19 patients while also addressing associated comorbid conditions.

SARS-CoV-2, the causative agent of COVID-19, was declared a pandemic by the World Health Organization. Given the absence of targeted therapeutics during the initial stages of the outbreak, research has focused on identifying potential inhibitors of the SARS-CoV-2 MPro, which plays a crucial role in viral replication. Computational studies have demonstrated that certain *W. somnifera* constituents exhibit strong inhibitory potential against MPro. Notably, molecular docking analysis revealed that Withanoside II (-11.30 kcal/mol), Withanoside IV (-11.02 kcal/mol), Withanoside V (-8.96 kcal/mol), and Sitoindoside IX (-8.37 kcal/mol) exhibited significant binding affinities [84]. Further 100 ns molecular dynamics (MD) simulation studies confirmed that Withanoside V demonstrated a strong binding affinity and stable hydrogen-bonding interactions with the active site of MPro, with a binding free energy score of (−87.01 ± 5.01 kcal/mol). Additionally, WS extracts contain withanolides and Withaferin A, both of which have demonstrated antiviral activity against SARS-CoV-2 [125, 126]. Studies have further highlighted the immunomodulatory potential of *W. somnifera*, suggesting its role in enhancing immune responses against COVID-19 and its variants [126, 127]. Furthermore, withanolides derived from *W. somnifera* have exhibited promising antiviral effects that may contribute to COVID-19 treatment [128]. Notably, withanone, another bioactive constituent, has demonstrated in vitro antiviral activity by inhibiting the SARS-CoV-2 MPro and the transmembrane protease TMPRSS2, both essential for viral entry and replication [70]. Despite these promising findings, further in vivo research and clinical trials are necessary to validate the therapeutic potential of WS and its bioactive compounds against SARS-CoV-2. A deeper understanding of its mechanisms of action, optimal dosages, and potential synergistic effects with existing antiviral therapies will be crucial for its clinical application.

### 6.8. Enhancement of Muscle Strength

Several investigations have been extensively conducted on WS for its effects on muscle strength. Research suggests that supplementation with ashwagandha root extracts (AREs) results in a significant improvement in muscle strength, size, endurance, and recovery in combination with resistance training [129–131]. In a recent study, a double-blind, placebo-controlled study showed that 28 days of 600 mg ARE markedly enhanced total quality of recovery (TQR) and reported sleep quality in female footballers, improving recovery indices relative to the placebo group [132]. Additionally, research involving 57 novice resistance trainers investigated the impact of ARE on muscular strength and hypertrophy. Participants were allocated to either a treatment group (300 mg administered twice daily) or a placebo group for a duration of 8 weeks. Strength was evaluated using 1-RM bench press and leg extension, and creatine kinase levels were assessed to gauge muscle recovery. The Ashwagandha group exhibited superior strength enhancements (bench press: +46.0 kg vs. +26.4 kg; leg extension: +14.5 kg vs. +9.8 kg) and muscular hypertrophy (arms: +8.6 cm; chest: +3.3 cm) relative to the placebo group. They exhibited less muscle damage, elevated testosterone levels, enhanced CAT activity, and a lower body fat percentage. The results were statistically significant (*p* < 0.05) [130]. WS enhances muscle strength, endurance, and recovery. A study on hockey players found that 500 mg WS root extract twice daily for 8 weeks significantly improved core muscle strength and stability (*p* < 0.02). WS also reduces muscle damage by lowering creatine kinase levels and increasing VO₂ max, enhancing aerobic endurance. Additionally, WS promotes lean muscle mass and strength through its adaptogenic effects on testosterone and stress modulation. These findings highlight WS as a natural ergogenic aid for enhancing athletic performance and muscle health [133]. A study involving elderly subjects (12–13 months, comparable to 60–65 human years) assessed the effects of *Withania somnifera* extract (WSE), protein supplements, whey protein, and resistance training on muscle health over 60 days. The treatments improved grip strength, increased biceps mass, and reduced glucose levels, inflammation (CRP, IL-6, TNF-*α*), oxidative stress, and apoptosis. WSE combined with protein produced the most significant benefits, enhancing muscular strength by increasing protein levels, antioxidant activity, and ATP availability [134]. These studies suggest that WS can be a valuable natural supplement for individuals looking to enhance their muscle strength and overall performance, especially when combined with a regular workout plan.

## 7. Application in Food

**Figure 6:**
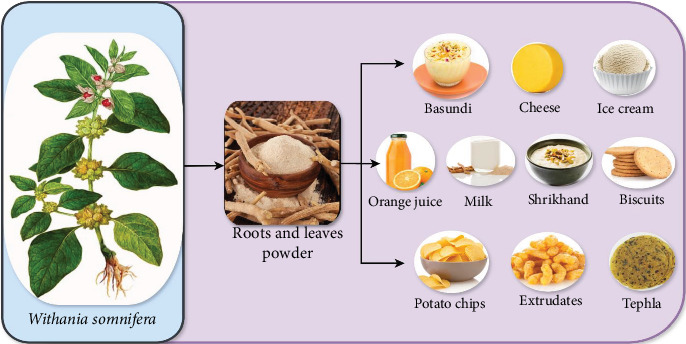
Incorporation of ashwagandha powder in developing various functional foods.

In recent times, there has been a growing awareness among consumers about the importance of diet and its effect on health [135]. This understanding has led to the development of various food products that address nutritional requirements for improved immunity and overall well-being. One approach is to include healthful components in familiar dishes. Adding plant-based components to ordinary dishes can make them more nutritious and healthier while maintaining their taste. Herbs such as ashwagandha offer great potential in developing new and healthy dishes [11]. Many experts are looking for methods to include ashwagandha, a famous Indian plant, into everyday dishes to increase their potential health benefits and enhance their nutritional value.  Figure 6 shows different functional foods that are incorporated with powdered extracts of ashwagandha.  Table 3 summarizes various functional foods incorporated with ashwagandha extract, detailing the extract type, associated therapeutic or nutritional benefits, and their current development or market status.

### 7.1. Shrikhand

Shrikhand, a traditional Indian subcontinental dish made from strained yogurt, has been enriched with ARE to create a functional food with enhanced nutritional and sensory properties [148]. Using response surface methodology (RSM) with a central composite rotatable design (CCD), the optimal levels of ARE, powdered sugar, and chakka were determined as 0.742%, 29.16%, and 69.48%, respectively. This formulation resulted in a product with 60.45% moisture, 15.92% fat, and significant antioxidant activity [44]. Additionally, another study evaluated the effect of ashwagandha powder in shrikhand by incorporating 40% cane sugar (by weight of chakka) and ashwagandha powder at three levels: 0.3% (T1), 0.5% (T2), and 0.7% (T3). The study assessed sensory attributes, microbial quality, and shelf life. T2 had the highest scores for color (7.98), flavor (8.11), and texture (8.11) on the 15th day, while T3 had the highest acidity (7.97 on the 7th day). T1 showed moderate sensory improvements, with a flavor score of (7.85) and texture score of (7.97) on the 15th day. Microbial analysis showed lower SPC in ashwagandha-treated samples, with coliforms undetectable after 22 days and reduced yeast and mold counts (8–10 cfu/g on the 30th day). The lactobacilli count increased over time, peaking at (23.6 × 10^6^ cfu/g) in T0 on Day 60. Shelf life improved, with T2 and T3 remaining acceptable for 52 days, T1 for 45 days, and T0 for 37 days [13]. Overall, these studies suggest that incorporating WS extract into dairy products not only improves their nutritional value but also extends shelf life due to the antioxidant properties of the extract.

### 7.2. Indian Flatbreads

Functional foods play a vital role in improving health, particularly in the management of diabetes [149]. WS is one of the well-known functional ingredients in traditional Indian diets, recognized for its antidiabetic properties [150]. Furthermore, a study was conducted on the impact of incorporating ashwagandha root powder into various food products on glycemic response. The study evaluated different concentrations (2%, 4%, 6%, and 8%) of ashwagandha in chapati, naan, and thepla, with 2% incorporation receiving the highest sensory acceptance. In a clinical assessment involving 30 healthy individuals, the GI of thepla (2%) was found to be 37.30, with a GL of 11.36, classifying it as a low-GI and medium-GL food [14]. Thus, ashwagandha root powder can be used to make low glycemic, palatable food products, which can be helpful in the management of diabetes. Such ingredients, when added to various Indian flatbreads, can generate several low glycemic foods, promoting health, enhancing nutritional profile, preventing diseases, and supporting overall health and well-being.

### 7.3. Potato Chips

To enhance the nutritional value and health benefits of potato chips, functional ingredients such as Andean potatoes and ashwagandha can be incorporated. Ashwagandha is well-known for its stress-reducing properties and immune-boosting effects [151]. Additionally, its inclusion enhances the overall value of potato chips. Andean potatoes serve as a valuable addition to healthy snacks like potato chips due to their high polyphenol content, which possesses antioxidant properties that may contribute to overall health and even promote tumor cell death [152]. Cold storage of potatoes increases reducing sugar levels, leading to browning and acrylamide formation, which negatively affects both the taste and nutritional quality of chips. A study investigated the use of WS *α*-amylase inhibitor (WASI) at 200 ppm for 30 min on potato slices. This treatment reduced browning by 60%, decreased residual amylase and polyphenol oxidase activity by 40%, and lowered sugar levels by 25%. Color analysis indicated improved whiteness and brightness, along with reduced yellowness. WASI-treated chips exhibited superior quality and nutritional value, effectively lowering acrylamide levels more than traditional blanching methods. Moreover, these chips retained twice the DW when fried, compared to blanched ones, outperforming synthetic inhibitors like acarbose and TAAI. This suggests that WASI is an ecofriendly and cost-effective alternative [15]. Incorporating Andean potatoes and ashwagandha into potato chips enhances their nutritional profile and health benefits. Ashwagandha not only reduces stress and boosts immunity but also improves chip quality, providing a natural and environmentally sustainable alternative to synthetic additives.

### 7.4. Extrudates

For the development of functional foods containing ingredients like ashwagandha, extrusion technology plays an important role. Studies have shown that the physical parameters of extrudates, such as sectional expansion index and mass flow rate, are influenced by factors like moisture content, barrel temperature, blend ratio, and screw speed [153]. Furthermore, the quality of extrudates is determined by molecular interactions among proteins, starch, lipids, and water during extrusion, affecting sensory quality, digestibility, and nutrient availability [138]. Additionally, extrusion cooking has been used to develop innovative products like cereal-based snacks and precooked breakfast cereals, meeting specific dietary needs and enhancing health benefits [154]. Therefore, the addition of ashwagandha into extruded functional foods can provide nutritional benefits and improve sensory and physical properties [155]. A study was conducted on the effect of antioxidants in developing fortified snacks with the addition of ashwagandha. The study shows that snacks fortified with ashwagandha have higher antioxidant content compared to the control [11]. The study highlighted that the extrusion cooking of these herbal snacks retained more bioactive compounds compared to the traditional cooking methods used for dried products. To increase its potential health benefits, this value-added processing method for incorporating herbal antioxidants requires more research and promises to be a highly promising field for industrial applications.

### 7.5. Basundi

In India, about 132.4 million tons of milk is produced annually, with nearly 50% of this milk production being converted into traditional Indian dairy products [156]. Dairy products are consumed all over the country and can be functionalized effectively. Moreover, developing carriers for herbs meets the demand of health-conscious consumers [157]. The flavonoids that are present in herbs aid in performing a wide range of biological functionalities. A large portion of the functional food market today comprises herbal supplemented functional foods [137]. Furthermore, basundi has been value-added by incorporating ashwagandha powder. Four formulations of basundi were prepared with ashwagandha at varying concentrations of 0% (P1), 0.20% (P2), 0.25% (P3), and 0.30% (P4) (w/w of milk). Among these, the basundi prepared with a 0.25% concentration of ashwagandha powder (P3) and 6% sugar with cardamom flavor 0.02% exhibited superior sensory attributes. The process has been standardized by incorporating mixed milk with a fat-to-SNF ratio of 0.5, 0.25% ashwagandha powder, 6% sugar, and 0.02% cardamom, scoring 90.13 out of 100 on the BIS sensory evaluation scale, indicating superior quality and high consumer acceptance. This finding added therapeutic benefits and modernized basundi by enhancing its commercial potential [158]. WS contains a wide range of bioactive compounds which are responsible for various biological activities. Incorporating ashwagandha powder into dairy products will not only enhance the nutritional profile but also increase the medicinal value.

### 7.6. Juices and Beverages

Beverages have become the most popular choice; they can easily meet consumer preferences in terms of size, shape, storage, and the inclusion of essential nutrients and bioactive compounds [159]. In recent decades, the functional beverage market has expanded significantly [160]. They are marketed for their various health benefits, such as gut protection, enhancement of cardiovascular function, enhancement of immune functions, aiding in weight management, and antiaging processes [161]. The diversity and specificity of such offers have courted an eclectic audience in search of health and well-being in every sip. A study was conducted on the development of a functional RTS beverage blend using ashwagandha WS and makoi (*Solanum nigrum*) separately with orange (*Citrus sinensis* L). The formulation comprised 85% orange juice, 10% sucralose, 0.1% citric acid, 6% of either herb, 0.05% carmoisine, and 0.05% propylene glycol, resulting in a TSS of 8.2°Bx. The blend was bottled, pasteurized, and stored at room temperature. The quality and stability attributes of ashwagandha fortified beverage and makoi fortified beverage were analyzed periodically for up to 3 months and compared with control orange juice. Both ashwagandha and makoi fortified beverages remained acceptable for 90 days when stored at room temperature. The findings show that the ashwagandha fortified blend beverage antioxidant activity is (899 ± 22.4*  μ*mol TE/100 g) while the antioxidant activity of makoi-fortified blend beverage is (750 ± 21.8*  μ*mol TE/100 g), proving effective in enhancing the nutritional quality of functional beverages [16]. The findings suggest that juices and beverages have become the most popular drinks, but they may not provide significant health benefits. Incorporating ashwagandha extract into juices and beverages will enhance the nutritional quality and functional properties.

### 7.7. Cereal and Bakery Products

Several scientific studies have focused on the evolution of baked goods as carriers for functional food products. Products range widely from biscuits, bread, cookies, cakes, dry crackers, and snack bars [162]. The popularity of these foods increases worldwide as consumers seek convenient, healthy snacks in line with their active lifestyles [163]. A wide range of products have been introduced to the market with different ingredients and formulations, merging the convenience of snacks with health and satiety. The rising demand for “on-the-go” products is induced by adapting consumption habits and diverse lifestyle choices [164, 165]. A study was conducted on the incorporation of ashwagandha for the development of three value-added products, namely, ashwagandha sweet and salty biscuit, ashwagandha churan ball, and ashwagandha beverage. These were prepared by incorporation of ashwagandha root powder, ashwagandha leaf powder, and ashwagandha root and leaf powder. Among the three products, only the ashwagandha biscuit was ranked higher in acceptability. This might be due to the bitterness of the leaves. The changes in sensory attributes of the products differed slightly during the 30-day storage period. Researchers developed an herbal-based functional food using ashwagandha leaf powder in another study. They found that the developed product had a higher nutritional value as compared to the control sample [166]. The ashwagandha-based cookies were a good source of several bioactive components that will help improve overall health and enhance potential health benefits [11]. Further studies were conducted on the development of the cereal-legumes incorporated biscuits using ashwagandha root powder at different proportions (3%, 4%, and 5%), and among these different concentrations, the best combination was found to be 5%. The findings concluded that treated biscuits were rich in energy, minerals, fibers, protein, and enhanced medicinal properties [142]. Ashwagandha fortification studies show that its inclusion might lead to increased nutritional and medicinal properties in cereal and bakery items making them functional foods despite challenges with bitterness from leaves, specifically in items like biscuits.

### 7.8. Ice Cream

Ice cream is defined as a dairy product characterized by a physicochemical composition, made by using both traditional and industrial methods. It contains ingredients such as milk, cream, sugar, stabilizers, and emulsifiers, along with fresh and dried fruits [167]. The enhanced versatility and widespread appeal of ice cream have prompted a rise in research efforts aimed at improving its functional characteristics [168]. The growing interest in functional foods has led to the incorporation of medicinal and nutraceutical ingredients into ice cream formulations. Studies have demonstrated that the addition of bioactive plant extracts and functional ingredients can enhance the nutritional and therapeutic potential of ice cream, making it a viable carrier for functional compounds [147]. Additionally, the incorporation of WS extract into ice cream formulations (T1, T0, T2, and T9) has been evaluated for sensory acceptance. Studies show that overall acceptability scores ranged from 6.80 to 8.74, with higher levels of ashwagandha (0.2%–1.0%) and strawberry pulp (10%–20%) reducing acceptability. The highest score was recorded for T1 (8.74), followed by T0 (8.38) and T2 (8.19), while T9 had the lowest (6.80). Notably, commercial production of ashwagandha-enriched strawberry ice cream could yield a 125% profit margin, highlighting its potential as a functional dairy product [169]. In research conducted by Vijay et al., the difference observed in the physicochemical properties of ice cream was due to the addition of ashwagandha and pineapple. Various levels of ashwagandha and pineapple were added and optimized based on sensory tests. Increased levels of pineapple raise moisture content, while ashwagandha powder has the opposite effect; protein levels exhibit a similar pattern, while fat content decreases with higher levels of both pineapple and ashwagandha; in contrast, pH levels decrease with higher levels of both ashwagandha and pineapple. The study concluded that good-quality ice cream can be prepared using fruit and medicinal plants [170].

The findings suggest that incorporation of ashwagandha into products like ice cream enhances its functional properties by influencing the physicochemical characteristics of the product, thereby supporting the development of nutritionally enriched and high-quality ice cream.

### 7.9. Milk

Milk is a nutrient-rich food, essential for human growth and development. India leads in global milk production with 155.54 million tons annually and a per capita availability of 337 g/day. About 55% of milk is processed into traditional dairy products, while 0.7% is used for ice cream production. Ice cream, a nutritious frozen dairy product, contains 4.9% protein, 13%–14% fat, and 20.3% carbohydrates, providing 214 kcal per 100 g [169, 171]. With increasing awareness of the connection between health and nutrition, people are now embracing new eating habits that encourage the consumption of healthy foods as opposed to fancy foods [172]. Recent research highlights the potential of ashwagandha in functional foods for sleep enhancement. A randomized, double-blind, controlled study evaluated the effects of 250 mL of lactose-free skimmed milk enriched with ashwagandha (250 or 600 mg), alone or with tryptophan (175 mg), on sleep quality in adults with sleep disturbances. Over 90 days, 52 participants were assigned to four groups, including a control of receiving nonenriched milk. Results showed significant improvements in sleep quality (*p* = 0.014) across all ashwagandha groups compared to the control, with the greatest benefits at 600 mg. While the ashwagandha-tryptophan combination did not outperform ashwagandha alone, reductions in insomnia severity and daytime somnolence were observed. No significant changes in anxiety or circadian rhythm were noted, and the formulations were well tolerated. These findings support the use of ashwagandha-enriched dairy products for sleep improvement [145].

A study was conducted on the effect of incorporating WS root powder on the properties of skimmed and standardized cow milk, including compositional, physicochemical, physical, functional, and sensory properties. It was observed that irrespective of the type of milk, total solids and carbohydrate percentages showed an increasing trend with higher levels of ashwagandha root powder, while a significant reduction in RCT was observed with increasing ashwagandha root powder percentages. Sensory scores decreased with increasing levels of ashwagandha root powder in milk, due mainly to the sedimentation effect [17]. The addition of WS root powder to cow milk resulted in a significant increase in the total solid and carbohydrate content with an appreciable reduction in RCT. However, higher doses might diminish sensory attributes due to sedimentation causing a trade-off between nutrient benefits and consumer acceptability.

### 7.10. Other Food Products

Additionally, several other studies have been investigated on the incorporation of ashwagandha powder into food to increase the functional properties and overall quality of the food. The incorporation of ashwagandha powder into quarg cheese enhanced the protein (13.47%), fat (10.89%), lactose (2.27%), acidity (0.62%), ash (1.46%), antioxidant activity (86.31% inhibition), and phenolic content (54.85 mg GAE/g) [173]. Supplementation of cereal-legume blend ladoo with ashwagandha not only enhanced shelf life by 30 days but also improved the nutritional profile. Increasing the level of ashwagandha enhanced the level of crude fat, crude fiber, total dietary fiber, and mineral content. The fruit extract of WS (0.5%) was incorporated into cheese, enhancing the resistance to lipid oxidation, reducing microbial contamination, improving sensory quality and storage stability, and boosting functionality of the cheese [136]. The addition of ashwagandha to milk helps in the treatment of stress-oriented hypertension [174]. Incorporation of ashwagandha into traditional products like Muruku, Chutney powder, Namakpara, Pappu chakalu, and Missi roti can enhance the functional properties as well as improve health [175]. Some traditional products like muruku, pappu chakalu, and namakpara are not good for health with prolonged consumption. By adding ashwagandha to these traditional products, it will enhance the functional properties and reduce cholesterol levels.

A study was conducted to evaluate the physicochemical properties, nutritional content, and sensory quality of low-fat sponge cakes incorporated with ashwagandha and giloy during storage. The addition of 2% ashwagandha powder and 5% giloy powder in sponge cake formulation resulted in improved sensory evaluations, with the tasting panel members expressing overall acceptability. During the 5-day storage period, protein, fat, carbohydrate, and energy content as well as TPC and antioxidant activity decreased. The study also highlighted the use of yogurt as a fat replacer in the low-fat sponge cake recipe, which enhanced its nutritional profile. The fortified sponge cakes retained their sensory quality and nutritional properties largely after storage, suggesting the possibility for developing functional and nutritious baked items enhanced with ashwagandha and giloy [176]. Burfi was incorporated with a blend of different herbs such as ashwagandha, shatavari, and tulsi in different ratios. The results suggested that there is a decrease in moisture content with increasing herb addition, while enhancement in total solids and protein content occurred. Fat and lactose content remained unchanged with herb addition. Ash content increased with higher herb addition rates. Overall, the addition of herbs influenced the chemical composition of burfi [177]. The addition of ashwagandha in the formulation of herbal ghee showed enhanced antioxidant properties [143]. Ashwagandha has been incorporated into functional fermented probiotics, prebiotics, and synbiotics from nondairy products [178]. The biotransformation capabilities of probiotics play a vital role in enhancing the bioactive components of medicinal and food homology (MFH) products such as ashwagandha through fermentation.

## 8. Safety and Toxicity of Ashwagandha

Ashwagandha has been recommended for numerous diseases since 1000 BC, as documented in Indian medicine texts such as *Charaka Samhita* and *Susruta Samhita* [19]. Toxicological tests have shown ashwagandha to be safe for treating both acute and chronic medical conditions [150]. It has been used safely across all ages and genders, including during pregnancy. Multiple in vitro and in vivo studies have examined ashwagandha's toxicology, confirming it as a healthy, safe, and digestible supplement when consumed in acceptable amounts [179].

Withaferin A, the primary bioactive compound in WS, possesses anticancer, antidiabetic, neuroprotective, hepatoprotective, and immune-modulatory properties [180]. Despite its limited oral bioavailability, Withaferin A has been found safe at 2000 mg/kg for acute toxicity and 500 mg/kg for recurrent toxicity [181]. KSM-66 Ashwagandha, a root extract produced using an aqueous-based extraction process, has been tested at doses of 500, 1000, and 2000 mg/kg body weight, showing no morbidity, mortality, adverse clinical signs, or significant effects on body weight, feed consumption, hematological, or biochemical parameters [182].

A 28-day subacute toxicity study found ARE (800 mg/kg) to be nontoxic, though prolonged exposure led to mortality, suggesting safety at 200–800 mg/kg for 28 days [183]. Another study on Withaferin A in mice at 2000 mg/kg for 28 days showed no observed organ damage, with a no observed adverse effect level (NOAEL) of 500 mg/kg when extrapolated to rats [181].

WS root extract has also been studied for its effects on sleeplessness and anxiety. Clinical trials demonstrated improvements in sleep onset latency, sleep efficiency, Pittsburgh Sleep Quality Index, and anxiety ratings after 10 weeks of treatment [184]. Additionally, WS root extract is rich in antioxidants, protecting the liver from CCl4 metabolism-induced free radical damage by increasing antioxidant enzyme levels. Toxicity studies have shown that the root extract does not cause mutagenic or genotoxic effects and is generally safe for animals [185]. Some studies reported minimal central nervous system depression and an increase in T4 levels, which normalized thyroid-stimulating hormone (TSH) in rats [186].

Preliminary MTT (3-[4,5-dimethylthiazol-2-yl]-2,5 diphenyl tetrazolium bromide) assays suggest that various WS components are nontoxic to different cell lines [187]. Research has also demonstrated the safety of WS extract in pregnant rats, showing no mortality, structural abnormalities, or developmental impairments [188]. In terms of sexual health, WS root extract has been investigated for its role in enhancing female sexual function, showing safety and efficacy in clinical trials [189].

For thyroid health, studies have found that WS root extract significantly improves thyroid hormone levels in hypothyroid patients after 8 weeks of treatment [190]. However, some reports indicate that commercial WS formulations have been linked to liver damage [191]. Excessive consumption can lead to a reduction in GSH levels, resulting in cytotoxicity and potential liver toxicity [192]. The growing market for herbal supplements necessitates continuous monitoring of their safety. Therefore, the research indicates that ashwagandha is found to be safe and may be further investigated for medicinal applications. No harm was reported in acute, subacute, and chronic toxicity trials, indicating a broad safety margin for ashwagandha.

## 9. Limitations and Future Studies

Despite the extensive research on WS, several limitations exist. The bioavailability of its bioactive compounds, particularly withanolides, remains a challenge, necessitating advanced delivery systems for improved absorption. Additionally, variations in extraction techniques, plant source, and processing methods affect the consistency of bioactive content, highlighting the need for standardized formulations. Quantitative studies on the concentration and stability of bioactive compounds in different food matrices are still limited. Future research should focus on high-performance liquid chromatography (HPLC), gas chromatography–mass spectrometry (GC-MS), and liquid chromatography–mass spectrometry (LC-MS) techniques to precisely quantify withanolides, flavonoids, and alkaloids in ashwagandha-enriched functional foods. Investigations on the effect of pH, temperature, and storage conditions on the degradation kinetics of these compounds would help optimize their stability and efficacy in food applications.

The incorporation of WS into functional foods presents promising opportunities, but several technological and sensory challenges need to be addressed. Future studies should explore the interaction of ashwagandha bioactives with dairy, bakery, and beverage formulations to ensure optimal stability and bioavailability. Additionally, sensory evaluation and consumer acceptance trials are essential to determine the ideal concentration of ashwagandha that maintains therapeutic efficacy while preserving taste and texture. Research on encapsulation techniques, such as nanoemulsions and spray-drying, can improve the retention of bioactive compounds during food processing and storage. Furthermore, shelf-life studies should be conducted to assess the long-term stability of ashwagandha-enriched food products, ensuring their commercial viability and nutritional effectiveness.

## 10. Conclusion

WS is recognized for its therapeutic importance and is extensively analyzed for its diverse bioactive compound characteristics. This review is aimed at methodically categorizing the reported bioactive compounds according to the group of secondary metabolites. This review also delves into various extraction methodologies and their effects on the resulting outputs. Additionally, since the era of Ayurveda, WS has been utilized in medicine traditionally. It exhibits a wide range of biological activities, such as anti-inflammatory, immunomodulatory, antimicrobial, cardioprotective properties, antioxidant activity, improvement of cognitive function, muscle strength, and inhibition of SARS-CoV-2; these properties are due to the presence of a wide range of bioactive compounds, mainly withanolides and other phytoconstituents. However, there are gaps and limitations in current extraction techniques, underscoring the necessity for more standardized methods and rigorous regulatory frameworks. It is important for comprehensive studies to be conducted on safety and efficacy before increasing its application to utilize fewer social resources. Every day, food is consumed to supply the body with necessary energy and nutrients and to combat diseases. Therefore, it is crucial to promote foods fortified with ashwagandha and its bioactive components. In the market, people prefer products with ashwagandha powder for their functional properties and potential health benefits. In connection with this, there is a need for standardization as well as the development of more value-added products around various parts of ashwagandha; although it possesses great therapeutic value, it requires further processing for its health advantages to be maximized. This review will serve as a valuable reference for future research in the field.

## Figures and Tables

**Table 1:** Withanolides and their derivatives isolated from various parts of the *Withania somnifera.*

**Sl. no**	**Plant parts**	**Bioactive compounds**	**Functions**	**Applications**	**References**
1.	Shoots	Withanolide A (2.88 mg/g DW) Withanolide B (1.48% mg/g DW) Withanolide D (0.30 mg/g DW) Withaferin A (3.79 mg/g) Withasomnillide Withasomniferanolide Somniferwithanolide Somniwithanolide Withanone (0.022 mg/g)	• The shoots of ashwagandha are believed to help in balancing hormones, specifically in relation to thyroid function and reproductive health. • It is used traditionally to boost energy levels, combat fatigue, enhance vitality, and improve overall well-being. • It helps to enhance memory.	• Fresh juice derived from WS shoots can treat chronic fever, by stimulating the immune system. • Drinking tea produced from WS shoots can help you relax, reduce anxiety, and sleep better.	[7, 22–24]

2.	Roots	Withanolide A (5.4 mg/g DW) Withanolide B (2.59 mg/g DW) Withanolide C (0.195%) Withanolide D (0.08%–0.11% in DW) Withanolide E (0.122%) Withaferin A (2.36 mg/g DW) Withanone (4.32 mg/g DW) 2,3-Dehydrosomnifericin 2,3 Dihydro withaferin A 27-Hydroxywithanolide A Withanolide dimer sulphide 27-Deoxy withaferin A 27-Hydroxy withanolide B Coagulin H Coagulin S Pseudotropine Isopelletierine 16b-Acetoxy-6,7a-epoxy-5-a-hydroxy-1-oxowitha2,17(20) 24-Trienolide 5,7a-epoxy-6a,20a-dihydroxy-1-oxowitha-2,24-dienolide Withasomniferol A Withasomniferol B Withanoside I (0.0020%) Withanoside II (0.012%) Withanoside III (0.0024%) Withanoside IV (0.048%) Withanoside V (0.017%) Withanoside VI (0.024%) Withanoside VII (0.0011%) Withasomniferol C 3*α*-tigloyloxtropine tropine, cuscohygrine, anaferine, anahygrine, mesoanaferine, choline, withanine, visamine, hentriacontane, ashwagandhanolide, *β*-sitosterol, and d-glycoside, Physagulin D and Coagulin Q Withanone (4.32 mg/g DW) Withanolide dimer sulphide Withaferin A (2.36 mg/g) 17-hydroxy-27-deoxy withaferin A, 17-hydroxy withaferin A, 1,2-Deoxy-withastranolide (1.12 mg/g) Withastramonolide, 5-dehydroxy withanolide-R, withasomniferin-A, 1-oxo-5b, 6b-epoxy-witha-2-ene-27-ethoxy olide, 2,3- dihydro withaferin A, 24,25-dihydro-27-desoxy withaferin A, 27-*α*-b-D-glucopyranosyl physagulin D, physagulin D, withanoside I–VII,27 O-b-D-glucopyranosylviscosalactoneB, 4,16-dihydroxy-5b,6b-epoxyphysagulinD, Viscosa, Lactone B, and diacetyl withaferin A	• It aids in reducing the debility associated with aging. • It helps to manage rheumatism. • It helps in relieving constipation. • It is used to treat goiter. • It is administered locally for cold, cough, painful swellings, ulcers, and carbuncles • It helps with flatulent colic. • It helps in managing insomnia. • It is used in the treatment of leucoderma. • It helps to strengthen and calm the nervous system. • The roots can enhance the number of white blood cells. • It helps to regulate blood sugar levels and aids in the treatment of weight loss.	• Root powder when given with milk acts as an effective tonic for children suffering from emaciation. • The root powder, when given with warm milk before bedtime, can help to reduce anxiety and stress due to its adaptogenic properties. • Consuming root extract with honey and warm water will enhance immunity and overall strength. • Blending the root powder with ghee and sugar can boost stamina and vitality, particularly during times of weakness and fatigue. • Taking root extract with Brahmi (Bacopa monnieri) and honey has been utilized for centuries to enhance memory, concentration, and cognitive function. • Ashwagandha root powder, combined with honey and milk, is believed to enhance fertility and reproductive health in both men and women.	[1, 7, 21, 25–33]

3.	Fruits (seeds and berries)	Linoleic acid (0.23%) Elaidic acid (0.01%) Withanolide D (0.22%) L-asparaginase Fatty acids Sterols 4-Deocywithaperuvin 14*α*,17*α*-Dihydroxywithanolide R Withanamide A Withanamide B Withanamide C Withanamide D Withanamide E Withanamide F Withanamide G Withanamide H Withanamide I Tocopherols Hydrocarbons (squalene), tetracosanoic acid (0.880%), and oleic acid (0.14%) 24,25-Dihydrowithanolide VI Iso-withanone 6*α*,7*α*-Epoxy1*α*,3*β*,5*α*-trihydroxy-witha-24-enolide	• The fruits are anthelmintic, and when combined with astringent and rock salt, it helps to remove white spots from the cornea. • The fruits are diuretic and are also used for treating chest ailments	• The seeds are used to thicken milk, and berries are used as a substitute for rennet, to coagulate milk in cheese making	[1, 7, 21, 25, 27, 29]

4.	Leaves	Withaferin A (1.35 mg/g) 17*α*-Hydroxy Withaferin A Witharistatin 2,3-Dihydro-3*β*-hydroxy withanone-3*β*-O-sulfate Withanolide A (350 *μ*g/g) Withanolide B (0.05 mg/g DW) Withanolide C Withanolide D (0.08%–0.11% DW) Withanolide E Withanolide F Withanolide G Withanolide H Withanolide I Withanolide K Withanolide L Withanolide M Withanolide N Withanolide O Withanolide P Withanolide Q Withanolide R Withanolide T Withanolide U Withanolide Y Withanolide Z Withanoside IV (0.04 ± 0.01 mg/g) Withanoside V (0.11 ± 0.04 mg/g) Withanoside VI Withanoside VII Withanoside VII Withanoside VII Withanoside IX Withanoside X Withanoside XI 12-Deoxy withastramonolide (0.07 ± 0.00 mg/L) Coagulin Q Withanolide Viscosalactone B Sitoindoside IX Physagulin D Deoxywithastramonolide (0.18 mg/g DW) 2,3-dihydrro withaferin A 27-desoxy-24,25-dihydro withaferin A 6*α*-chloro-*β*,17*α*-dihydroxy withaferin A Withanone (1.312%) 6*α*-chloro-5*β*-hydroxy withaferin A 3-methoxy-2,3-dihydro withaferin A 2,3-diehydrosomnifericin 27-hydroxy withanolide B	• The leaves are anthelmintic (Kills intestinal worms) and recommended for fever, wounds, and painful swelling. • The paste made from the leaves is applied locally to eradicate lice on the body. • Leaf juice is useful in conjunctivitis.	The leaves are also extensively used at home on the form of herbal tea	[7, 21, 26, 27]

5.	Bark	Somnifera withanolide Withanolide Somniwithanolide Withasomniferanolide Somniferanolide Withasomnilide		Bark decoction is taken to treat asthma and applied locally for bed sores.	[27, 34]

**Table 2:** Key alkaloids and flavonoids isolated from various parts of the *Withania somnifera.*

**Sl. no**	**Plant parts**	**Alkaloids**	**Flavonoids**	**Extraction method**	**Extraction condition**	**References**
1.	Roots	Coniine (0.012%) Hyoscine (0.015%) Lobeline (0.018%) Ephedrine (0.025%) Solanidine (0.018%) Withasomine Somniferine Somniferinine Isopelletierine propanone Anaferine Anahygrine Pseudotropine Iron-pseudotropine Withanine 1 (Ganguly et al.) Oxacyclohexadecane-2,13-dione,13-oxime Ashwagandhine Scopoletin 2,4-Imidazolidiendione1-(5-nitro-2-furanyl) methylene Tropine Nicotine Choline Berberine Anahygrine Cuscohygrine Harmane Harmine Caffeine Isopelletierine Noscapine Papaverine Pseudotropine Sedridine Theobromine Theophylline 3*α*-tigloyloxytropane Yohimbine Withanine Somine Withananine Pseudo-withanine Tropine Pseudo-tropine, 3-a-gloyloxytropane, Cuscohygrine Andanahydrine	Catechin Hyperoside Rhamnetin Rutin (0.134%) Kaempferol (0.195%) Myricetin Quercetin (0.149%) Dihydroxykaempferol Querceti-3-rutionside Aesculentin Quercetin 3-O-robinobioside-7-O-glucoside (0.157%)	Reflux Ultrasound-assisted solvent extraction (USAE) Microwave-assisted solvent extraction (MASE) Maceration Exhaustive extraction	Reflux: solvent–methanol/water; temperature: 50°C–80°C; time: 2–3 h USAE: solvent–ethanol/water; frequency: 40 kHz; time: 30–60 min MASE: solvent–ethanol; temperature: 60°C; time: 5–20 min Maceration: solvent–water/ethanol; time: 24 h	[7, 21, 22, 26, 27, 45–48]

2.	Fruits	Withanamide A Withanamide B Withanamide C Withanamide D Withanamide E Withanamide F Withanamide G Withanamide H Withanamide I	Catechin Kaempferol Rutin Naringenin Naringin Kaempferol 3-robinobioside-7-glucoside Quercetin-3-rutinoside-7-glucoside	Maceration Reflux: mechanical stirring: liquid–liquid partitioning	Maceration: solvent–ethanol; temperature: 40°C–60°C; time: 24 h Reflux: solvent–methanol; time: 3 h	[7, 27, 45, 49–51]

3.	Berries	Isopellertierine Anaferine	Apigenin Apigenin-7-O- *β*-D-glucopyranoside Rutin Kaempferol Catechin	Super critical CO2 extraction	Pressure: 450/80 bar; temperature: 40°C; time: 22 h	[7, 52, 53]

4.	Leaves	Vasicine (0.014%) Tropine Nicotine Tisopelletierine Pseudotropine Anaferin Withasomine 3-Tropyltigloate Pseudowithanine Cuscohygrine 3*α*-Tigloyloxtropine Hygrine Withasomine Dl-isopelletierine Measoanaferine Withanine Somniferine Hentriacontane Withananine Visamine Ashwagandhine	Catechin (0.102%) 6,8-Dihydroxykaempferol 3-rutionside Quercetin-3-Rutinoside-7-Glucoside Rutin	Maceration: liquid–liquid partitioning Soxhlet extraction Microwave-assisted extraction (MAE) Subcritical extraction	Maceration: solvent–ethanol/methanol; temperature: 50°C–70°C; time: 6–12 h Soxhlet: solvent–hexane; cycles: 10–15; time: 8 h MAE: solvent–ethanol; temperature: 60°C; time: 5–10 min Subcritical extraction: temperature: 150°C–200°C; pressure: 100 bar; time: 20–30 min	[21, 22, 26, 54]

**Table 3:** Different functional foods incorporated with ashwagandha extract.

**S. No**	**Food product**	**Ashwagandha extract type**	**Therapeutic/nutritional benefit**	**Status**	**References**
1	Kalari cheese	Fruit extract (0.5%)	Improved microbial stability, antioxidant benefits, oxidative stress reduction	Under development	[136]
2	Indian flatbreads (Chapati, Naan, and Thepla)	Root powder	Lowers glycemic index, suitable for diabetes management	Under development	[14]
3	Potato chips	*α*-Amylase inhibitor extract	Reduces acrylamide formation, enhances antioxidant content	Under development	[15]
4	Functional RTS beverages	Extract (WS + makoi + orange juice)	Boosts immunity, rich in antioxidants	Under development	[16]
5	Milk-based functional beverage	Ashwagandha root extract	Increases total solids and carbohydrate content, improves RCT	Under development	[17]
6	Basundi (Indian dessert)	Ashwagandha powder	Enhance sensory and nutritional profile	Under development	[137]
7	Herbal extrudates (snacks, cereal-based foods)	Root extract	Rich in antioxidants, improves functional properties	Under development	[138]
8	Herbal supplements (capsules/tablets/powders)	Standardized root/leaves extract	Stress reduction, cognitive enhancement, immune modulation	Commercialized	[139]
9	Ashwagandha-infused chocolates	Root extract	Adaptogenic properties, reduces stress	Commercialized	[140]
10	Ashwagandha Tea/herbal infusions	Leaves/root powder	Reduces anxiety, improves relaxation	Commercialized	[141]
11	Ashwagandha-infused cereal-legume based ladoo	Root powder (3%–5%)	Increase fiber, fat, and mineral content; improves shelf life (30 days); provides curative effects	Under development	[142]
12	Ashwagandha-enriched shrikhand	Root extract (0.742%)	Improves antioxidant activity (DPPH 81.4%, ABTS 73.25%); increases total phenolic content (53.68 mg/100 g GAE); improves sensory attributes	Under development	[44]
13	Ashwagandha-enriched herbal ghee	Alcoholic extract (0.5%)	Enhanced sensory properties provide antioxidant and therapeutic properties.	Under development	[143]
14	Ashwagandha-enriched strawberry ice cream	Root powder	Increased antioxidant capacity improves texture and nutritional profile	Under development	[144]
15	Ashwagandha-fortified milk with tryptophan	Root extract (250 mg & 600 mg)	Improves sleep quality, reduces insomnia severity	Under development	[145]
16	Ashwagandha-infused sandesh	Root extract (2%–3%)	Improves sensory attributes, enhances antioxidant properties	Under development	[146]
17	Ashwagandha-fortified ice cream	Root powder	Improves hardness, adhesiveness, and nutritional profile	Under development	[147]

## Data Availability

Data sharing is not applicable to this article as no new data were created or analysed in this study.
